# Coking wastewater treatment plant as a sources of polycyclic aromatic hydrocarbons (PAHs) in sediments and ecological risk assessment

**DOI:** 10.1038/s41598-020-64835-2

**Published:** 2020-05-12

**Authors:** Jundong Chen, Jianbo Liao, Chaohai Wei

**Affiliations:** 1 School of Environment and Energy, South China University of Technology, Guangzhou, 510006 P.R. China; 2 Research Center for Eco-Environmental Engineering, Dongguan University of Technology, Dongguan, 523808 China

**Keywords:** Environmental sciences, Environmental social sciences, Risk factors

## Abstract

The spatial and temporal distribution of polycyclic aromatic hydrocarbons (PAHs) was investigated in sediments of Maba River, a major tributary of Beijiang River (South China). A total of 13 samples from Maba River and its tributary, Meihua River, were analyzed for 16 PAHs. The total concentration of 16 PAHs (ΣPAH) in high and low water period ranged between 47.61 to 25480.98 ng g^−1^, with a mean concentration of 4382.98 ng g^−1^, and 60.30 to 15956.62 ng g^−1^ with a mean concentration of 3664.32 ng g^−1^, respectively. Three-ring and four-ring PAHs were the dominant species. It was concluded that a pattern of pyrolytic input as a major source of PAHs in sediments through the molecular ratio method for the source identification, such as HMW/LMW PAHs, Flu/(Flu+Pyr), IcdP/(IcdP+BghiP) and BaA/(BaA+Chr). It is suggested that the pollution emission from the iron and steel plant might be the most important sources of PAHs into Maba River water system. The threat of PAHs contamination to biota of the river was assessed using effect range low (ERL) and effect range median (ERM) values, which suggested that PAHs in Maba River and its tributary had already caused ecological risks.

## Introduction

Polycyclic aromatic hydrocarbons (PAHs) are an important class of persistent organic pollutants (POPs) widely distributed in environment, including soils, atmosphere, water body and sediments^1,2^. PAHs are a class of diverse compounds that consist of two or more fused aromatic rings. PAHs have been regarded as priority pollutants by both the US Environmental Protection Agency (EPA) and the European Union (EU) due to their carcinogenicity, mutagenicity, and toxicity on both ecosystem and human health^3^. PAHs are mainly emitted from anthropogenic activities such as coal combustion, industrial manufacturing, waste incineration, and power generations^4–6^. Anthropogenic PAHs can be released into all the environment^7^. Industrial and municipal WWTPs are identified as point sources for PAHs released into the aquatic environment^8,9^. It is necessary to investigate characteristics and pollution of the sediments to deal with problems related to aquatic environments^10^.

Due to the hydrophobic and lipophilic property, PAHs can be absorbed to suspended particles and subsequently deposit in sediments during wastewater treatment^11,12^. Sedimentary PAHs tend to accumulate to high concentrations can create toxicity to benthic organisms and to pelagic organisms. PAHs (with high concentration (more than 1000 μg g^−1^) was detected in some of the most highly industrialized and urbanized locations^13–15^. The developed techniques make the analysis of sediment PAHs possible, which can serve as useful index of the contamination level of PAH input released to the aquatic environment^16–18^.

Maba River, located in Qujiang District, Shaoguan City, Guangdong Province, China, belongs to the Beijiang River water system and is the left bank tributary of Beijiang River. Maba River originates from Huangmaozhang, the junction of Qujiang District and Wengyuan Country, flows to the northwest through Maba town. After the import of a tributary, named Meihua River in Caoxinping, Maba river flows finally in Longtouzhai into the Beijiang River. The total length of the main stream is 43.235 km and the basin area is 345 km^2^. Meihua River, which originates from Beizishan, Maba town, is a secondary tributary of Beijiang River. The length of Meihua River is 26.4 km and the rainwater harvesting area is 80.9 km^2^.

The Maba River plays an important role in the enrichment and migration of pollutants in its basin: on the one hand, it receives the pollutants from the concentrated source (the mine in the upstream area, the companies, such as Shaoguan Steel Plant and Shaoguan Smeltery) and the scattered source (the living area and the agricultural belt on both sides of Maba River); on the other hand, it become a pollutions-source of its final influx, Beijiang River, the main source of drinking water in northern Guangdong province.

Among the two sources of pollution, concentrated industrial pollutions, especially in the smelting and coking industries, generates a large amount of waste water, waste gas and waste residue in the production process, and contains a large amount of toxic organic matter and heavy metals. The potential hazard of toxic organic pollutants is very serious for the surrounding residents. With the rapid development of economy, industry and urbanization, especially the large-scale enterprises, such as Shaoguan Steel Plant and Shaoguan Smeltery that listed in the top 500 Chinese industrial enterprises, caused more pollution to the river. Many anthropogenic pollutants possessed the capability to accumulate in the sediments^19^. Considering the health of local residents and the quality of drinking water, the degree of river pollution and the control of various pollution points and emissions source should be systematically evaluated. However, so far, there is little information regarding the spatial distribution and contamination level of PAHs in the sediment of the Maba River. It’s essential to determine the sources to assess the significant risk of contamination by PAHs^20^.

In order to comprehensively understand the pollution characteristics and source contribution of sediment PAHs in the Maba River, and provide basic data for environmental management and water pollution prevention and control in Beijiang River Basin, this paper takes the sediments of Maba River as the research object and investigate the contamination level and distribution of the polycyclic aromatic hydrocarbons through sampling and investigation, and the ratio method have been employed to identify their possible sources and assess the potential risks to the environment.

## Materials and methods

### Sample collection

**Figure 1:**
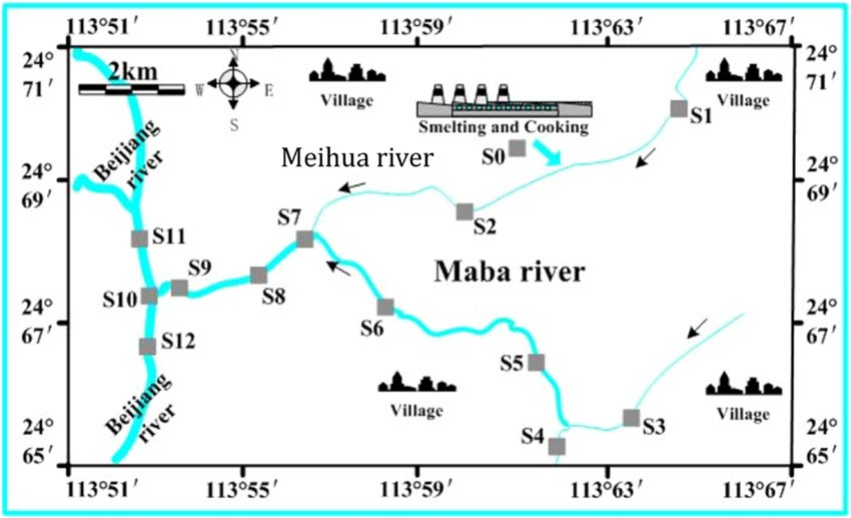
Map of sampling sites.

The sample collection time of the surface sediments of the Maba River is from March 2013 to January 2014. The sampling sites are shown in Fig. 1. A total of 13 sampling sites along the Maba river and its tributaries. The surface sediment sample (0–20 cm) was collected using a cylindrical sediment core sampler (inner diameter 6 cm, length 100 cm), 2 kg per sampling point, and then placed in a clean polyethylene sealing bag. After collection, the sediments were stored in 4 °C ice box and transited to the laboratory for experiment. In the laboratory, the sediments were kept in the refrigerator at −20 °C before analysis. All sediment samples were freeze-dried and then ground uniformly, and stored at −18 °C for analysis.

### Chemical reagents

Chemicals, suppliers, and purities are listed in the Supplementary Data, Text S1.

### Instrumental detection

Quantification of PAHs was performed using a Thermo Scientific TRACE GC Ultra gas chromatography with a HP-5MS capillary column (30 m × 0.25 mm i.d. × 0.25 µm film thickness). Instrumental conditions were described in Text S2.

### Quality assurance and quality control

Quantification was performed using a five-point calibration curve established using hexane-based internal standard for each individual PAH. The R^2^ values of the PAH calibration curves were all greater than 0.99. The relative percent analysis difference of paired duplicate samples was <15%. The instrument detection limits (IDL) for each PAH were 0.1–2 ng g^−1^. The limit of quantitation (LOQ) of the method is determined by based on Signal-to-Noise. The signal measured at the lowest point of the calibration curve was 10 times greater than noise in repetition. The data with concentrations below LOQ was not included for the calculation of mean values. The average recoveries for all the sludge samples were 58.7 ± 8.7% for naphthalene-d8, 74.6 ± 9.1% for acenaphthene-d10, 86.7 ± 8.7% for acenaphthene-d10, 96.8 ± 10.1% for chrysene-d12, and 96.0 ± 9.7% for perylene-d12.

### Data statistics and pollution source analysis, potential ecological risks assessment

After analyzing the PAHs content of each sampling sites, the data was compared with other rivers in China and abroad to determine the pollution level of the sediments of the Maba River. By analyzing the spatial and temporal distribution of PAHs pollution, and then combining the ratio method, the source of PAHs pollution and the impact of smelting and coking enterprises in the basin of Maba River was determined. And the potential ecological risks of PAHs in sediments would be evaluated. This would provide a basis for river management in the Maba River and Beijiang River Basin and pollution sources control for smelting and coking enterprises.

## Results and discussion

### Concentrations of PAHs in sediments

The concentrations of 16 EPA-PAHs in surface sediments from the Maba River and its tributary, Meihua River in high water period and low water period are presented in Table S1 and Table S2. As shown in Table S1 and Table S2, in the high water period, the ΣPAHs values ranged from 47.61 (S4) to 25480.98 (S2) ng g^−1^, with a mean concentration of 4382.98 ng g^−1^, and in the low water period, the ΣPAHs values ranged from 60.30 (S11) to 15956.62 (S2) ng g^−1^ with a mean concentration of 3664.32 ng g^−1^. There’s one point which needs attention that the median concentration of ΣPAHs was much lower than the mean both in the high and low water period, which indicated that some sites contained relatively higher concentrations. It is obvious that the site here is S2. Different from other sampling sites, S0 is located at the beginning of the sewage outfall of Shaoguan Steel Plant Company, all industrial and domestic sewage flows through this channel into the Meihua River. The tail water after coking wastewater treatment usually contains higher concentrations of PAHs, the values of ΣPAHs in S0 reached 173111.20 ng g^−1^. The inflow of the tail water with high PAHs concentration from the upstream of S2 into the Meihua river directly led to a 1000-times increase in the values of PAHs from S1 to S2. This analysis showed that the comprehensive impact of confluence of the smelting and cooking tail water and the Meihua River is huge.

To better understand the magnitude of PAHs concentrations in sediments from Maba River, comparison of PAHs concentrations with previous research were conducted (Table S4). As shown in Table S4, the concentrations of ΣPAHs in sediments from Maba River was relatively high compared to other Chinese studied rivers. Among the Chinese rivers, the level of ΣPAHs in sediments from Maba River was much higher than those of Lijiang River (160–602 ng g^−1^)^21^, Qiantang River (91.3–614.4 ng g^−1^)^22^, upper reach of Huaihe River (95.2–877.5 ng g^−1^)^23^, Minjiang River (112–877 ng g^−1^)^24^, middle and lower reaches of Yellow River (16–1358 ng g^−1^)^25^, Huangpu River (313–1707 ng g^−1^)^26^, Haihe River (445–2185 ng g^−1^)^27^, Yangtze River (72.4–3995.2 ng g^−1^)^28^, Pearl River (597–10811 ng g^−1^)^29^. It was only lower than Haihe River (Tianjin) (775–255372 ng g^−1^)^27^ and Tianjing Rivers (787–1943000 ng g^−1^)^30^. Compared to other rivers around the world, the level of ΣPAHs in sediments was also much higher^31–33^ and comparable to Yamuna River, India. The Shaoguan Steel Plant and Shaoguan Smeltery have been the most important Iron and Steel plant in the Maba River Basin for the past several decades. Contrast the situation of the rivers in Tianjin, Tianjin’s smelting and chemical industry is highly developed. The Tianjin Iron and Steel Group is located beside the Haihe River and larger than the Shaoguan Steel Plant. In the basin of Pearl River (the level of ΣPAHs was also high) there is also a large smelting company Zhugang. Therefore, the pollution discharge from Shaoguan Steel Plant and Shaoguan Smeltery is the reason for the higher concentrations of PAHs than other rivers.

### Spatial distributions of PAHs in sediments

As described above, in the Maba River and its tributary, the highest levels of PAHs were observed in S2 site located in the Meihua River. The upper reach of S2 site is the sewage output of the Shaoguan Steel Plant, which was between S1 and S2. When the Meihua River flows through the Shaoguan Steel Plant, with the inflow of the tailwater, the concentration of ΣPAHs gradient increased from 116.73 (S1) to 25480.98 (S2) ng g^−1^ in the high water period, and in the low water period it increased from 74.02 (S1) to 15956.62 (S2) ng g^−1^. The results showed that the Shaoguan Steel Plant was responsible for the high level of PAHs in S2.

In the main river of Maba River, whether in the high or low water period, the concentration of ΣPAHs in S6 site is higher than in S5 site, which is the upper reach. The Maba town is located between the two sites, so the discharge of urban sewage is the main reason. In the lower reach of the Maba River, the second increase of ΣPAHs concentration (S6 to S7) probably resulted from the inflow of Meihua River, S7 is located at the downstream of the confluence of Maba River and Meihua River. Therefore, it may be mainly ascribed to the PAHs inputs from Meihua River, which possessed the highest PAHs levels in this studied river (Table S1 and Table S2). Moreover, more PAHs in river could be easily absorbed to the particles, which resulted in the accumulation of PAHs in sediment. Site S9 is located near an industrial area, and the third increase of ΣPAHs concentration (S8 to S9) probably because the discharge of industrial wastewater. The reason of the increase of ΣPAHs concentration from S11 to S10 is the same as those from S6 to S7.

### Temporal distributions of PAHs in sediments

The PAH concentration in high water period was lower than in low water period dilution of contaminants by large volume of water. The coefficient of divergence (CD) was employed to study the similarity/dissimilarity of the PAH profiles in both periods in order to investigated the PAH sources in different periods^34,35^. The CD is self-normalizing and can be calculated from short-term measurements or long-term averages. The CD is defined as formula (1)^36^:

1 $$CD=\sqrt{\frac{1}{16}\mathop{\sum }\limits_{i=1}^{16}{\left(\frac{{x}_{if}-{x}_{ij}}{{x}_{if}+{x}_{ij}}\right)}^{2}}$$

Where 16 is the number of the each PAH species, ${x}_{if}$ is the average fraction (percentage) of the *i*th individual PAH species in the low water period, and ${x}_{ij}$ is the average fraction (percent) of the *i*th each PAH species in the high water period, and. If the average fraction of each PAH in the two periods is similar, the CD value approaches to 0; if the average fraction of the each PAH species in the two periods is quite different, the CD value approaches to 1. Similar PAH profiles may indicate similar sources. After calculation, the CD values for the Maba River and its tributary is 0.61, suggesting that less dissimilarity in the two periods. Hence, the PAHs might be attributed to similar pollution sources between different periods. In addition, in the low period, the river is stagnant and the flow velocities are very slow. This also accelerated the precipitation of particles that adsorbs PAHs in the water. In high water period, the high flow rate of the river water re-suspends surface sediments, leading to the decrease in total concentration of pollutants^37^.

The difference of PAH concentration in the two periods was shown in Table S3. In the low water period, the Σ PAH concentration ranged from 60.30 (S11) to 15956.62 (S2) ng g^−1^, with the mean value of 3664.32 ng g^−1^. In the high water period, the Σ PAH concentration ranged from 47.61 (S4) to 25480.98 (S2) ng g^−1^ with an average concentration of 4382.98 ng g^−1^. PAH concentration in high water period was higher than in low water period.

The concentration of PAHs in river sediments was generally affected by water flow. The continuous ad-sorption of PAHs in rivers with high level of PAHs leads to an increase in PAHs content in sediments, but the level of PAHs was reduced because of desorption by the addition of water with low concentrations of PAHs. Therefore, in general, the dilution of the river water flow during the wet season will result in a significant decrease in the PAHs concentration in sediment. By comparison of the PAHs concentrations in two periods in Table S1 and Table S2, the reason for above anomaly is obvious. The PAHs concentrations in sediments from S0 (173111.20 ng g^−1^) in the high water period is much higher than those (116925.70 ng g^−1^) in the lower water period, this means that more contaminants may be discharged during the high water period. The effect of the contaminants increase exceeds the dilution of water. The values of ΣPAHs in S3, S4, S5 and S6 in the dry period is visibly higher than those in the high water period and not affected by the PAHs from S0 area, this is in accordance with our previous analysis and the reports for the sediments of the Yamuna River in India^38^ and Gao-ping River in Taiwan^37^. Moreover, the median values of PAHs in Table S3 are much useful than the mean, It avoids the effect of extreme value (S2) on the data analysis.

Based on the analysis of the concentration distribution of PAHs in the Maba River and its tributaries, it is found that the PAHs content in the sediments of the Maba River would be affected by the season before the convergence with the Meihua River, which is in line with the general rule. However, due to the influence of the tail water discharged from the S0 site, the PAHs level in the sediment of the Meihua River has completely depended on the concentration of PAHs in the tail water, and is not affected by the season, and ultimately affects the Maba River after the convergence, and even Beijiang River.

### Composition and sources identification of PAHs in sediments

**Figure 2:**
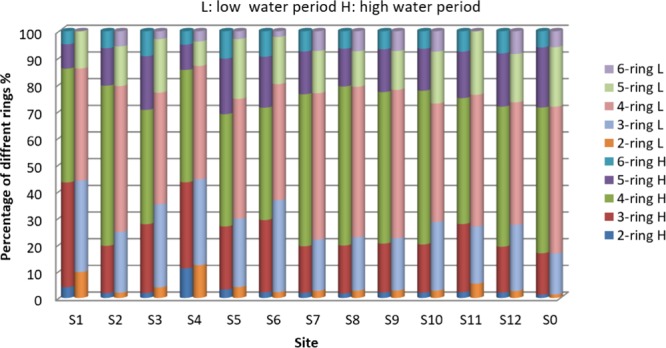
Composition of PAHs in sediment from Maba River in high and low water period.

**Figure 3:**
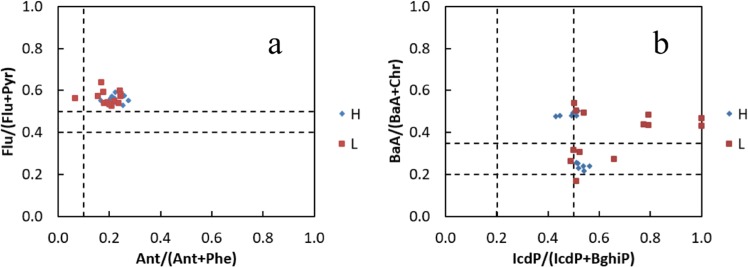
(**a**) Ratios of Ant/(Ant+Phe) vs. Flu/(Flu+Pyr) and (**b**) BaA/(BaA+Chr) vs. IcdP/(IcdP+BghiP).

**Figure 4:**
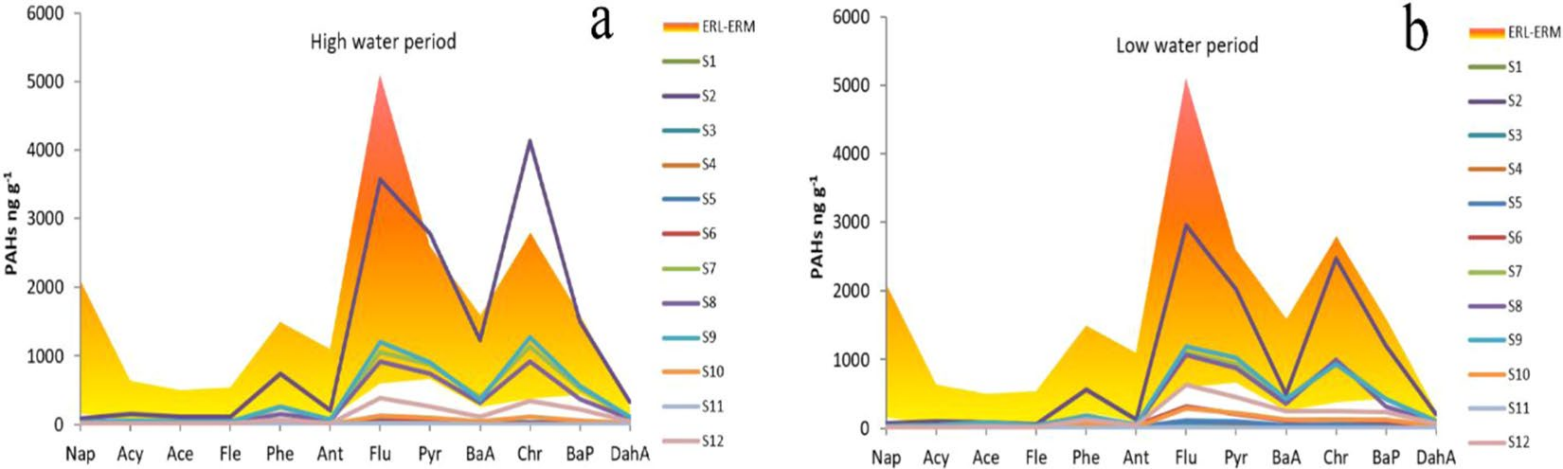
Comparison between PAHs in sediments and ERL and ERM values (**a**) high water period, (**b**) low water period.

In order to distinguish the PAHs sources, the levels of PAHs with different molecular weights were commonly performed^39^. The 16 PAHs were divided into five groups based on the number of aromatic rings (two-ring, three-ring, four-ring, five-ring and six-ring). The levels of individual PAHs in sediments from the Maba River in high and low water period was shown in Fig. 2. As presented in Fig. 2, four-ring PAHs (50.55% of ΣPAHs in high water period, 48.53% in low water period, on average) was most abundant and three-ring PAHs ranked second, which accounted for 22.75% and 25.13% of ΣPAHs in high and low water period. Five-ring and six-ring occupied 16.52% and 7.32% of ΣPAHs in high water period, 17.38% and 4.79% of ΣPAHs in low water period on average, respectively, while two-ring PAHs possessed the least proportions. The fraction of PAHs in both periods was similarity indicated that the dominant sources for PAHs in sediments of Maba River were similar in different period (Figs. 3 and 4).

PAHs are mainly produced from incomplete combustion of fossil fuel (pyrogenic), and the discharge of petroleum and its products (petrogenic)^17^. The PAH composition pattern is beneficial to track the contaminant source, and understand the fate and transport of PAHs in a complex environment^34,40^. Molecular indexes based on ratios of selected PAHs were employed to distinguish PAHs from pyrogenic and petrogenic sources. For example, HMW/LMW PAHs, Flu/(Flu+Pyr), IcdP/(IcdP+BghiP), BaA/(BaA+Chr) were applied for PAHs source identification.

In this study, high-molecular-weight (HMW) from four-ring to six-ring PAHs (>70%) were predominated in the sediments from Maba River. Pyrogenic sources were enriched in HMW PAHs via combustion processes. Petrogenic sources were dominated by LMW PAHs through the releasing of fuel oil or light refined petroleum products. Thus, the HMW/LMW ratio may be used to determine the sources of PAHs^41–43^. the LMW/HMW ratios were determined to estimate the sources of the PAHs in sediment samples from the Maba River (Table S5). As illustrated in Table S5, all the sampling sites with relatively high HMW/LMW ratios (1.25–4.99) indicated that pyrolytic sources played a major role.

As shown in Table S5, several ratios of Ant/(Ant+Phe), Flu/(Flu+Pyr), BaA/(BaA+Chr), and IcdP/(IcdP+BghiP) have been developed for the analysis of possible sources^41,44^. In the present study, the Ant/(Ant+Phe) ratios ranged from 0.15 to 0.25 in high water period and 0.07 to 0.25 in low water period, while the Flu/(Flu+Pyr) ratios ranged from 0.53 to 0.64 in both periods. Only the Ant/(Ant+Phe) ratio of S1 is less than 0.1 (Figs. 3a and 4). The IcdP/(IcdP+BghiP) ratios in high and low water period ranged from 0.43 to 0.66 and from 0.49 to 1.00, respectively. The BaA/(BaA+Chr) ratios ranged from 0.22 to 0.50 in high period and from 0.17 to 0.64 in low water period, some ratios ranged between 0.2 to 0.35, but most of which is greater than 0.20 (Fig. 3b). This indicated that there was mainly pyrolytic source of PAHs in sediments in this basin.

According to the above results, the values of some ratios were not in agreement among them in some cases (Fig. 3). Thus, the total index of these rations of specific PAHs compounds is useful for interpreting compositions and inferring possible sources. The total index as the sum of single index respectively normalized for the limit value (low temperature source-high temperature source) was employed to characterize the source of PAHs due to the different and occasional source of PAHs in a matrix^45,46^. As shown in formula (2):

2 $$Total\,index=\frac{\frac{Ant}{Ant+Phe}}{0.1}+\frac{\frac{Flu}{Flu+Pyr}}{0.4}+\frac{\frac{IcdP}{IcdP+BghiP}}{0.2}+\frac{\frac{BaA}{BaA+Chr}}{0.35}$$

The PAHs originated by high temperature processes (combustion) when the total index was greater than 4. The lower values, less than 4, indicated low temperature source, such as petroleum product. The total indexes of the sediments from Maba River were greater than 6, confirming that the PAHs were from pyrolytic source.

Based on those data, it could be concluded that PAHs in the sediments from Maba River mainly generated from coal, grass, wood and petroleum combustion. This result was corresponded to the sample’s surroundings. S5 and S6 were located around Maba town, the main energy source for urban residents and cars is the burning of combustible materials, which also affects the time distribution of PAHs in sediments. The lower temperature in Guangdong in dry season leads residents to need more fuel for combustion. S2 was located at the Shaoguan Steel Plant, which has a coking plant. It has been reported that the pollution of PAHs around a coking plant was from the pyrolysis of coal^35,47,48^.

The values of isomeric ratios obtained from wastewater and wastewater sludge of coking plant are presented in Table S6. These results indicated that PAHs in the coking wastewater and wastewater sludge still possessed the characters of combustion and pyrolysis, which was similar with coal, like the previous results of sediments from Maba River. Higher concentrations of PAHs were present in the final effluents of coking wastewater treatment processes due to the high hydrophobic property of PAHs^49^. The coking plant effluents were discharged to the Maba River and diluted therein, this is the reason why the values of PAHs in S0 and S2 are particularly higher than others.

### Potential ecological risks of PAHs in sediments

The effect range low (ERL) and effect range median (ERM) were applied to evaluate the potential toxicity effects of sediments in Maba River and its tributary. These two guideline values were helpful for addressing sediment quality issues with a ranking of low to high impact values and provided qualitative guidelines for the work of effectively protection of the aquatic-environment^50^. The ERL and ERM values are developed to define chemical concentration ranges (Table S7) that are rarely, occasionally, or frequently associated with adverse biological effects. The concentration of PAHs is below the ERL value would be expected to rarely have adverse biological effects, while the PAH concentrations higher than ERM suggest a probable toxic response in aquatic organisms, And PAHs levels that between ERM and ERL are considered to have moderately adverse effects on aquatic organisms.

As for some compounds, the ERL and ERM values have been compared to PAH concentrations in sediments from Maba River (Table S7). As Seen from Table S7, the results exhibited that the risk level was relatively high in Maba River and its tributary, while most sites presented environmental risks by PAHs. The highest ΣPAHs content was found at S0 site (173111 ng g^−1^ in high water period and 116926 ng g^−1^ in low water period) and was much higher than the corresponding ERM value (44792 ng g^−1^), suggesting that the frequent adverse ecological effects were expected. The PAHs from S0 also affects its downstream. As shown in Figs. 3a and 4 in both period, most PAH level of sediments from its downstream (S2, S7, S8 and S9) in Maba River is over ERL, which indicated that adverse environmental effects occurred occasionally or frequently. For sampling sites S3, S4, S5 and S6, Individual PAH concentration did not exceed their respective ERL values. It suggested that the biological risk of this section of Maba River was relatively low.

## Conclusions

This paper provided data on the spatial, temporal distributions and sources analysis of PAHs in sediments from Maba River and its tributary (Meihua River). The potential environmental health risks were also evaluated. It showed PAHs pollution in the Maba River basin was associated with sewage input from industrial activities. The spatial distribution of PAHs in sediments from the main stream implied that the input of the tributary (Meihua River) played an important role for Maba River. The data provided evidence that the areas adjacent to the Shaoguan Steel Plant could be regarded as hot spot areas of PAH pollution for Maba River. Compared to some other rivers around the world, the PAH concentration in the sediments were at a high level. The PAH concentration was higher in high water period than in low water period, which indicates that the change of pollutant discharge in the Shaoguan Steel Plant area has become the decisive factor for the change of PAHs level in the Maba River Basin. Three-ring and Four-ring PAHs were major species in sediment samples. According to the principal component analysis, PAHs in the sediment were mostly originated from pyrogenic due to the combustion of coal and biomass. The both periods should be accountable for the same contamination sources. This also conformed to the characteristics of the surrounding environment. The tail water discharged from the Shaoguan Steel Plant has caused PAHs pollution in the Maba River Basin and formed ecological risks. The sediments after the convergence still had a high concentration of PAHs, which has already caused harm to the water quality of the Beijiang River. It was necessary to make effective efforts for source management and pollution control. This study provided useful baseline reference concentration for PAHs monitoring programs in the future.

## Supplementary information

Supplementary information.
