# Supplementary material for: Coking wastewater treatment plant as a sources of polycyclic aromatic hydrocarbons (PAHs) in sediments and ecological risk assessment
Source: Sci Rep. 2020 May 12;10:7833. doi: 10.1038/s41598-020-64835-2 (PMC7217903; doi:10.1038/s41598-020-64835-2)
Supplement: Supplementary file 1 — Supplementary information. [file 41598_2020_64835_MOESM1_ESM.pdf]

Supplementary Material for

Coking wastewater treatment plant as a sources of polycyclic aromatic hydrocarbons (PAHs) in sediments and ecological assessment

Jundong Chen[a], Jianbo Liao[b], Chaohai Wei[a*]

[a] School of Environment and Energy, South China University of Technology, Guangzhou 510006, P. R. China

[b] Research Center for Eco-Environmental Engineering, Dongguan University of Technology, Dongguan 523808, China

* Corresponding author at School of Environment and Energy, South China University of Technology, Guangzhou 510006, P. R. China

Phone: +86 20 39380588

Email address: cechwei@scut.edu.cn

Text S1

Standard PAHs including naphthalene (Nap), acenaphthylene (Acy), acenaphthene (Ace), fluorene (Fle), phenanthrene (Phe), anthracene (Ant), fluoranthene (Flu), pyrene (Pyr), benzo[a]anthracene (BaA), chrysene (Chr), benzo[b]fluoranthene (BbF), benzo[k]fluoranthene (BkF), benzo[a]pyrene (BaP), indeno-[1,2,3-cd]pyrene (IcdP), dibenzo[a,h]anthracene (DahA), benzo[g,h,i]perylene (BghiP) and deuterated PAHs standards containing naphthalene-D8, acenaphthene-D10, phenanthrene-D10, chrysene-D12, and perylene-D12 were obtained from Dr. Ehrenstorfer GmbH (Augsburg, Germany). The organic solvents, dichloromethane (DCM), n-hexane, cyclohexane, acetone, and methanol, used for sample extraction and purification procedures were HPLC grade and purchased from CNW Technologies GmbH (Duesseldorf, Germany).

Anhydrous sodium sulfate (analytical grade; Xilong Scientific Co., Ltd, Shantou, China) and alumina (100-200 mesh) (Tianjin Kemiou Chemical Reagent Co., Ltd., Tianjin, China) were baked in a furnace oven at 450 °C for 4 h and then kept in a sealed desiccator prior to use. Cu was obtained from Damao Chemicals Co., Ltd (Tianjin, China). Water was prepared from a Barnstead Nanopure UV D11911 ultrapure water system (Thermo Scientific, USA) with a specific resistivity of 18.2 MΩ cm.

Text S2

Instrumental conditions: injector temperature, 260 °C; Sample (1 μL) was injected in splitless mode; Helium was used as the carrier gas at a constant flow rate of 1.2 mL/min. The oven temperature was ramped from 60 °C (1 min) to 160 °C (8 min) at 10 °C/min and then to 300 °C at 6 °C/min and held for 8 min. Ion source temperature was 230 °C. The MSD was operated in the selected-ion-monitoring (SIM) mode. Data acquisition and processing was controlled by Thermo Xcalibur software. Chromatographic peaks of samples were identified by mass spectra and by comparison with the standards.

Table 1 PAHs concentrations in sediments form Maba River, high water period (ng g^{-1}, dry weight)

	2 Rings				3 Rings			4 Rings					5 Rings			6Rings	
**PAHs**	**Nap**	**Acy**	**Ace**	**Fle**	**Phe**	**Ant**	**Flu**	**Pyr**	**BaA**	**Chr**	**BbF**	**BkF**	**BaP**	**DahA**	**IcdP**	**BghiP**	**ΣPAHs**
**S0**	190.0	1150	450.2	320.7	2920	590.3	23400	13200	9440	24600	32000	15300	14000	4750	20300	10500	173111.20
**S1**	n.d	n.d	2.47	2.23	11.06	1.98	32.82	24.55	6.69	7.24	7.55	3.73	6.05	n.d	4.62	5.74	116.73
**S2**	88.98	155.2	109.1	105.6	742.6	210.7	3561	2773	1225	4122	5501	1676	1503	332.8	1751	1624	25480.98
**S3**	n.d	3.35	1.88	1.86	23.42	6.18	68.75	53.35	28.52	28.08	36.76	16.87	31.47	7.39	38.02	35.75	381.65
**S4**	n.d	1.09	2.58	1.60	4.22	1.11	10.04	8.73	3.03	3.31	2.88	2.08	2.71	n.d	1.82	2.41	47.61
**S5**	n.d	2.87	2.03	0.59	8.72	2.25	30.19	23.47	11.92	12.95	16.92	8.08	13.32	4.24	18.75	17.88	174.18
**S6**	n.d	1.73	2.28	1.26	18.38	3.67	45.52	37.08	16.96	17.47	22.8	10.99	19.44	4.59	23.68	23.93	249.78
**S7**	25.43	52.41	44.28	29.59	243.3	68.84	1049	870.4	392.9	1138	1521	526.1	522.9	108.6	621.3	594.8	7808.85
**S8**	18.56	37.36	28.51	20.91	152.1	57.64	912.7	742.5	314.1	919.8	1377	370.4	364.8	82.78	437.8	411.4	6248.36
**S9**	26.89	58.26	45.09	41.21	264.3	69.69	1199	896.5	352.1	1272	1707	530.5	561.3	113.4	671.8	568.4	8377.44
**S10**	3.21	5.05	4.23	4.56	23.98	8.36	121.8	90.48	37.36	117.5	182.5	64.09	56.85	11.21	66.14	56.54	853.86
**S11**	n.d	0.93	n.d	0.74	3.45	1.17	15.22	13.63	5.69	6.15	7.63	3.59	5.87	1.92	5.75	5.96	77.70
**S12**	10.21	16.32	15.23	14.58	72.03	20.57	384.7	264.1	108.4	341.9	552.4	192.7	215.2	42.11	297.4	230.8	2778.65

n.d: not detected

Table S2 PAHs concentrations in sediments form Maba River, low water period (ng g^{-1}, dry weight)

	2 Rings				3 Rings			4 Rings					5 Rings			6Rings	
**PAHs**	**Nap**	**Acy**	**Ace**	**Fle**	**Phe**	**Ant**	**Flu**	**Pyr**	**BaA**	**Chr**	**BbF**	**BkF**	**BaP**	**DahA**	**IcdP**	**BghiP**	**ΣPAHs**
**S0**	128.8	780.5	308.4	283.5	1945	397.5	15690	8976	6295	16879	21335	10832	9335	3188	13552	7000	116925.70
**S1**	3.22	n.d	2.78	1.17	7.89	0.56	16.94	13.23	4.55	6.02	4.68	2.58	3.32	n.d	7.08	n.d	74.02
**S2**	68.34	102.8	86.74	51.74	562.4	121.3	2952	2029	495.8	2478	2497	1224	1202	212.7	959.1	913.7	15956.62
**S3**	2.67	5.64	3.03	8.42	54.86	10.19	91.13	68.53	34.95	44.86	42.22	19.54	34.44	14.9	51.47	14.9	501.75
**S4**	0.72	2.07	3.32	3.16	5.68	1.51	17.15	15.46	5.32	4.55	3.52	3.24	2.12	1.92	2.94	2.92	75.60
**S5**	5.34	9.89	4.62	5.54	40.8	8.97	110.2	94.84	44.32	58.02	57.48	26.18	49.83	18.51	71.78	18.51	624.83
**S6**	3.36	11.73	5.04	10.51	130.7	41.35	312.6	209.2	117.2	124.6	109.8	51.31	101.5	30.38	117.1	30.38	1406.76
**S7**	24.59	49.45	81.12	30.91	191.8	45.87	1102	935.3	429.9	982.8	1063	418.1	421.9	116.9	567.3	516.9	6977.84
**S8**	19.38	38.03	74.96	42.43	167.1	46.91	1070	875.4	355.8	999.1	1027	353.4	312.1	81.6	460.7	481.6	6405.51
**S9**	26.61	49.66	84.02	38.27	163.9	50.54	1195	1021	428.1	931.2	1151	459.7	421.2	93.15	533.2	533.1	7179.65
**S10**	6.61	9.66	14.02	10.27	63.92	20.54	295.5	221.5	128.1	131.2	115.1	59.77	121.2	33.15	133.2	113.1	1476.84
**S11**	3.05	n.d	n.d	0.16	2.29	0.58	10.09	8.84	5.37	6.15	6.41	2.97	5.21	n.d	9.18	n.d	60.30
**S12**	13.22	20.34	30.53	20.51	126.6	41.22	632.2	456.2	253.2	251.2	395.3	128.7	231.1	65.34	289.9	276.5	3232.06

n.d: not detected

Table S3 Concentrations of 16 PAHs in sediments S1-S12 from Maba River (ng g^{-1}, dry weight)

PAHs	High water period			Low water period		
	Range	Mean	Median	Range	Mean	Median
Nap	n.d - 88.98	14.44	1.61	0.72 - 68.34	14.76	5.98
Acy	n.d - 155.20	27.88	4.20	n.d - 102.80	24.94	10.81
Ace	n.d - 109.10	21.47	3.41	n.d - 86.74	32.52	9.53
Fle	0.59 - 105.60	18.73	3.40	1.17 - 51.74	18.59	10.39
Phe	3.45 - 742.60	130.63	23.70	2.29 - 562.40	126.50	95.26
Ant	1.11 - 210.70	37.68	7.27	0.56 - 121.30	32.46	30.88
Flu	10.04 - 3561.00	619.23	95.28	10.09 - 2952.00	650.40	304.05
Pyr	8.73 - 2773.00	483.15	71.92	8.84 - 2029.00	495.71	215.35
BaA	3.03 - 1225.00	208.56	32.94	4.55 - 495.80	191.88	122.65
Chr	3.31 - 4122.00	665.53	72.79	6.02 - 2478.00	501.48	127.90
BbF	2.88 - 5501.00	911.29	109.63	3.52 - 2497.00	539.38	112.45
BkF	2.08 - 1676.00	283.76	40.48	2.58 - 1224.00	229.12	55.54
BaP	2.71 - 1503.00	275.24	44.16	2.12 - 1202.00	242.16	111.35
DahA	n.d - 332.80	59.09	9.30	n.d - 212.70	55.71	31.77
IcdP	1.82 - 1751.00	328.17	52.08	2.94 - 959.10	266.91	125.15
BghiP	2.41 - 1624.00	298.13	46.15	n.d - 913.70	241.80	71.74
ΣPAHs	47.61 - 25480.98	4382.98	617.76	60.30 - 15956.62	3664.32	1441.80

Table S4 Total PAHs concentration ranges and mean values in sediments of different rivers around the world

Site	n	ΣPAHs (ng g^{-1}) Range	Mean	References
Lijiang River, China	16	160 - 602	337	(Zhang et al., 2013)
Qiantang River, China	15	91.3 – 614.4	313.3	(Chen et al., 2007)
Huaihe River (upper reach), China	16	95.2 – 877.5	370.8	(Feng et al., 2012)
Minjiang River, China	16	112 - 877	433	(Zhang et al., 2003)
Yellow River (middle and lower reaches), China	16	16 - 1358	182	(Sun et al., 2009)
Huangpu River China	16	313 - 1707	1154	(Liu et al., 2009)
Haihe River, China	16	445 - 2185	964	(JIANG et al., 2007)
Yangtze River (Wuhan), China	16	72.4 – 3995.2	1334.5	(Feng et al., 2007)
Pearl River, China	16	597 - 10811		(Mai et al., 2002)
Haihe River (Tianjing), China	16	775 - 255372	27074	(JIANG et al., 2007)
Tianjing Rivers, China	16	787 - 1943000	10980	(Shi et al., 2007)
Gomti River, India	16	5.2 – 3722.9	697.2	(Malik et al., 2011)
Yser River, France	16	490 – 4160	1950	(Sanctorum et al., 2011)
Hyeongsan River, Korea	16	5.30 - 7860		(Koh et al., 2004)
Upper-Scheldt River, France	16	1800 - 8910	4870	(Sanctorum et al., 2011)
Yamuna River, India	16	4500 - 23530		(Agarwal et al., 2006)

n: Number of PAHs compounds analyzed in each study

Table S5 Characteristic values of selected molecular ratios for pyrolytic and petrogenic origins of PAHs

Ratio	Pyrolytic origin	Mixed origin	Petrogenic origin	This study		References
				H	L	
HMW/LMW	>1.0		<1.0	1.31-4.96	1.25 - 4.99	(Wang et al., 2006)
Ant/(Ant+Phe)	>0.1		<0.1	0.15- 0.25	0.07 - 0.25	(Soclo et al., 2000)
Flu/(Flu+Pyr)	>0.40		<0.40	0.53-0.64	0.53- 0.64	(Yunker et al., 2002)
BaA/(BaA+Chr)	>0.35	0.20-0.35	<0.20	0.22-0.50	0.17 - 0.64	(Yunker et al., 2002)
IcdP/(IcdP+BghiP)	>0.20		<0.20	0.43-0.66	0.49 - 1.00	(Yunker et al., 2002)

H: high water period, L: low water period

Table S6 Characteristic values of selected molecular ratios for pyrolytic andpetrogenic origins of PAHs, coking plant

Ratio	Pyrolytic origin	Mixed origin	Petrogenic origin	Coking plant	
				Wastewater (Zhang et al., 2013)	Wastewater sludge (Zhang et al., 2012)
Ant/(Ant+Phe)	>0.1		<0.1	0.12	0.12
Flu/(Flu+Pyr)	>0.40		<0.40	0.60	0.57
BaA/(BaA+Chr)	>0.35	0.20-0.35	<0.20	0.56	0.47
IcdP/(IcdP+BghiP)	>0.20		<0.20	0.55	0.56
Total index	>4			7.10	6.79

Table S7 Comparison of some PAHs in sediments from Maba River to their respective ERL and ERM (ng g^{-1}, dry weight)

PAHs	ERL	ERM	This study		Sites (S)					
					N of < ERL		S of ERL - ERM		S of >ERM	
			H	L	H	L	H	L	H	L
Nap	160	2100	n.d - 89	0.7 - 68	12	13	S0	-	-	-
Acy	44	640	n.d - 155	n.d - 103	9	9	S2 S7 S9	S2 S7 S9	S0	S0
Ace	16	500	n.d - 109	n.d - 87	8	7	S2 S7 S8 S9 S0	S2 S7 S8 S9 S12 S0	-	-
Fle	19	540	0.6 - 106	1.2 - 52	8	7	S2 S7 S8 S9 S0	S2 S7 S8 S9 S12 S0	-	-
Phe	240	1500	3.4 - 743	2.3 - 562	9	11	S2 S7 S9	S2	S0	S0
Ant	85	1100	1.1 - 211	0.6 - 121	11	11	S2 S0	S2 S0	-	-
Flu	600	5100	10 - 3561	10 - 2952	8	7	S2 S7 S8 S9	S2 S7 S8 S9 S12	S0	S0
Pyr	665	2600	8.7 - 2773	8.8 - 2029	8	8	S7 S8 S9	S2 S7 S8 S9	S0 S2	S0
BaA	261	1600	3.0 - 1225	4.6 - 496	8	8	S2 S7 S8 S9	S2 S7 S8 S9	S0	S0
Chr	384	2800	3.3 - 4122	6.0 - 2478	8	8	S7 S8 S9	S2 S7 S8 S9	S0 S2	S0
BaP	430	1600	2.7 - 1503	2.1 - 1202	8	10	S2 S7 S9	S2	S0	S0
DahA	63	260	n.d - 332	n.d - 213	8	7	S2 S7 S8 S9	S2 S7 S8 S9 S12	S0	S0
ΣPAHs	4022	44792	48 - 173111	60 - 116925	8	8	S2 S7 S8 S9	S2 S7 S8 S9	S0	S0

H: high water period, L: low water period
